# The Effectiveness of Zero-Profile Cages Versus Traditional Bone Graft Placement With Plate Fixation in Anterior Cervical Discectomy and Fusion for Single- and Two-Level Cervical Prolapsed Intervertebral Discs: A Comparative Prospective Study

**DOI:** 10.7759/cureus.102033

**Published:** 2026-01-21

**Authors:** Zia ur Rehman, Bilal Khan, Muhammad Aamir, Bashir Ullah, Syed Jawad Ahmad

**Affiliations:** 1 Neurosurgery, Medical Teaching Institution (MTI) Lady Reading Hospital, Peshawar, PAK

**Keywords:** anterior cervical discectomy and fusion (acdf), anterior cervical plate, iliac crest bone graft, interbody cage, one-level disc cervical pathology, prolapsed intervertebral disc (pivd)

## Abstract

Introduction

Anterior cervical discectomy and fusion (ACDF) is considered the gold standard procedure for fixation of cervical disc degenerative disease. However, the optimal construct remains debated between zero-profile cages and bone graft with anterior plate fixation.

Objective

This study aims to evaluate the clinical and radiological outcomes of zero-profile cages with standard bone graft and plate fixation in single- and two-level cervical prolapsed intervertebral disc surgery.

Methods

This prospective observational study was conducted at the Neurosurgery department after enrolling patients aged 18-70 years with symptomatic cervical disc disease refractory to ≥6 weeks of conservative management who underwent ACDF either with a zero-profile titanium cage (Group A) or with autologous iliac crest bone graft and anterior cervical plate fixation (Group B). Standard surgical technique (Smith-Robinson approach) and uniform postoperative protocols were used. Clinical outcomes (Neck Disability Index (NDI) and Visual Analogue Scale (VAS)) and radiological fusion were assessed preoperatively and at 12 months.

Results

A total of 103 patients were included in the study (Group A: 57; Group B: 46). At 12 months, significant improvement in NDI was observed in both groups (Group A: 21/57 (36.8%) had improved scores vs. 3/57 (5.3%) worsened; Group B: 17/46 (37.0%) improved vs. 3/46 (6.5%) worsened; both p < 0.01). VAS scores also improved significantly (Group A: 23/57 (40.4%) improved; Group B: 18/46 (39.1%); p < 0.01). Fusion was achieved in 51/57 (89.5%) in Group A and 39/46 (84.8%) in Group B (p = 0.301). Dysphagia with plate fixation was 3/46 (6.5%) and with zero-profile cages was 4/57 (7.0%). No significant difference in re-operations was noted.

Conclusion

Both techniques demonstrated comparable functional outcomes and fusion rates. However, zero-profile cages were associated with a significantly lower re-operation rate compared with bone graft and plate fixation, indicating a potential advantage in reducing revision surgery.

## Introduction

Cervical degenerative disc disease (CDDD) is an inevitable outcome of aging, marked by the gradual reduction in disc height and flexibility resulting from degeneration. These degenerative alterations may result in the production of bone spurs, disc herniation, and spinal canal stenosis [1]. Worldwide, neck discomfort is exceedingly common, with approximately 222.7 million instances and an age-standardized prevalence of 2,696.5 per 100,000 individuals [2,3]. Cervical radiculopathy is caused by nerve root compression, leading to pain and neurological symptoms in the arm and hand, whereas cervical myelopathy results from spinal cord compression, typically causing symptoms in both the upper and lower limbs and, in severe cases, bowel and bladder dysfunction. The prevalence of cervical spine pathology has increased, with younger individuals exhibiting progressively intricate instances [4].

Anterior cervical discectomy and fusion (ACDF) is the conventional surgical intervention for cervical disc herniation unresponsive to conservative therapy. This anterior approach facilitates the excision of the degenerative disc, alleviation of neural compression, restoration of disc height, and stabilization of the cervical spine [5-7]. Traditional ACDF employing a cage with anterior plating has shown elevated fusion rates; nevertheless, it is linked to problems including dysphagia, soft tissue irritation, and neighboring segment degeneration [8-10].

The creation of zero-profile devices, known as stand-alone or anchoring cages, aims to resolve these issues. These implants incorporate a cage with internal fixation facilitated by locking screws put into neighboring vertebral bodies [6,11]. Zero-profile cages have been shown to offer sufficient biomechanical stability in single-level ACDF, while also reducing operative duration, minimizing soft tissue damage, and lowering the risk of postoperative dysphagia and adjacent-level disease compared with traditional plate-and-cage constructs [12-14].

## Materials and methods

This prospective observational cohort study was conducted from April 2022 to May 2024 at the Neurosurgery Department, Lady Reading Hospital, Peshawar, Pakistan, a tertiary care teaching hospital, following approval from the institutional ethics review board (IRB No: 08.1/LRH/MTI). All participants provided written informed consent prior to enrollment.

Between April 2022 and May 2024, 127 patients with symptomatic single- or two-level CDDD, refractory to a minimum of six weeks of conservative treatment, were assessed for eligibility. A total of 24 patients were excluded: 11 had previous cervical spine surgery, five had traumatic cervical spine injury, three had active infection, two had malignancy, and three declined participation. The remaining 103 patients (aged 18-70 years) were enrolled and allocated to treatment groups based on surgeon preference and implant availability after preoperative discussion regarding the benefits and risks of each technique. Group A (n=57) received ACDF with a zero-profile cage and screw fixation, while Group B (n=46) received ACDF with autologous iliac crest bone graft and anterior cervical plate fixation. Sample size calculation, assuming a clinically meaningful difference of 10 points in the Neck Disability Index (NDI), a standard deviation of 15, a power of 80%, and an alpha of 0.05, required a minimum of 36 patients per group; we enrolled 103 patients to account for a potential 15% loss to follow-up.

All surgeries were performed by experienced spine surgeons (with a minimum of five years' experience in cervical spine surgery) using the standard Smith-Robinson anterior approach, with uniform perioperative protocols and rehabilitation guidelines for both groups.

Clinical outcomes were evaluated using the NDI and Visual Analogue Scale (VAS) for neck pain, documented at baseline and 12 months postoperatively [15,16]. The NDI is a validated 10-item questionnaire with scores ranging from 0 (no disability) to 50 (complete disability). Neck pain intensity was measured using a 10-cm horizontal VAS, where 0 represents "no pain" and 10 represents "worst imaginable pain." Clinical outcomes were assessed by independent neurosurgery residents not involved in surgical procedures at preoperative baseline, the immediate postoperative period, and at six weeks, three months, six months, and 12 months. Outcome assessors were not blinded to treatment allocation due to the observational study design and the visible radiographic implant differences.

Radiological fusion was assessed using dynamic lateral cervical radiographs at 12 months and classified using the Bridwell fusion grading system [17]: Grade 1 (fused with remodeling and trabeculae), Grade 2 (graft intact, not fully remodeled), Grade 3 (graft intact with obvious lucency), and Grade 4 (fusion absent with resorption/collapse). Grades 1 and 2 were considered successful fusion. Radiological assessments were performed independently by two experienced spine surgeons; consensus was reached through discussion in cases of disagreement. Inter-rater reliability showed substantial agreement (Cohen's kappa = 0.78).

Postoperative complications, including dysphagia, wound infection, neurological decline, hoarseness, adjacent segment degeneration, and re-operation necessity, were documented. Dysphagia severity was graded as mild (occasional difficulty with solids), moderate (frequent difficulty requiring dietary modification), or severe (requiring medical intervention). Wound infection was defined as purulent drainage, positive wound culture, or clinical signs requiring antibiotic therapy. Adjacent segment degeneration was defined as radiological evidence of new or progressive degenerative changes at levels immediately adjacent to the fusion site.

Efforts to address bias included standardized surgical technique, uniform perioperative protocols, validated assessment tools, and consistent follow-up schedules. However, selection bias may have occurred due to non-randomized allocation based on surgeon preference. Lack of blinding could introduce detection bias for subjective outcomes. Confounding by unmeasured variables (bone quality, anatomical variations, surgeon experience) remains a concern. Preplanned subgroup analyses evaluated outcomes separately for single-level and two-level ACDF procedures.

Statistical analysis was performed using IBM SPSS Statistics for Windows, Version 23 (Released 2015; IBM Corp., Armonk, New York). Descriptive statistics summarized demographics, surgical levels, and outcomes as numbers and percentages (n, %) for categorical variables and mean ± standard deviation for continuous variables. Within-group changes in NDI and VAS scores were evaluated using the Wilcoxon signed-rank test. Between-group comparisons for continuous outcomes used the Mann-Whitney U test, while categorical outcomes (fusion grades, re-operations, complications, and adjacent segment degeneration) used the chi-square test. Effect sizes were reported as mean differences with 95% confidence intervals for continuous outcomes and risk ratios with 95% confidence intervals for categorical outcomes. All 103 patients completed a 12-month follow-up with no missing data. Statistical significance was defined as p < 0.05.

## Results

The study included 103 patients who were evenly distributed between a zero-profile cage group (Group A) and a bone graft with plate group (Group B). Both groups were similar in terms of baseline characteristics, including mean age, gender distribution, rates of smoking, comorbidities, and the proportion of single- and two-level surgical procedures. Details are given in Table 1 below.

**Table 1:** Descriptive Characteristics of Both Groups

Variable	Group A (n=57)	Group B (n=46)	p-value
Age (years), mean ± SD	48.8 ± 8.5	49.3 ± 9.1	0.925
Gender			0.992
Male	25 (43.9%)	20 (43.5%)	
Female	32 (56.1%)	26 (56.5%)	
Smoking			0.443
Yes	3 (5.3%)	4 (8.7%)	
No	54 (94.7%)	42 (91.3%)	
Comorbidities			0.333
Yes	8 (14.0%)	4 (8.7%)	
No	49 (86.0%)	42 (91.3%)	
Level Operated			0.814
Single Level	42 (73.7%)	33 (71.7%)	
Two Levels	15 (26.3%)	13 (28.3%)	

In Table 2, both treatment groups demonstrated significant improvements in NDI and VAS scores. In the zero-profile cage group, approximately one-third of patients improved, while over half showed no change, and only a small proportion worsened; the differences were statistically significant. A similar pattern was observed in the bone graft with the plate group, with comparable rates of improvement and stability, also reaching statistical significance. Overall, both interventions yielded meaningful functional and pain-related improvements, with no major differences in the proportion of patients improving between the groups. P-values were calculated using the Wilcoxon signed-rank test, and a p<0.05 was considered significant.

**Table 2:** Wilcoxon Signed-Rank Test for NDI and VAS (Within Groups) NDI: Neck Disability Index; VAS: Visual Analogue Scale

Outcome	Group	Negative Ranks (Improved)	Positive Ranks (Worsened)	Ties	Total	Z	p-value
NDI	Group A	21	3	33	57	-3.644	<0.001
	Group B	17	3	26	46	-3.128	0.002
VAS	Group A	23	4	30	57	-3.580	<0.001
	Group B	18	2	26	46	-3.010	0.003

At the 12-month follow-up (Table 3), both groups demonstrated comparable outcomes, with no statistically significant differences in median NDI or VAS scores between the zero-profile cage and the bone graft with plate groups, indicating that both techniques were equally effective in the long term. P-values were calculated using the Mann-Whitney U test, and a p<0.05 was considered significant.

**Table 3:** Mann-Whitney U Test Between Groups (NDI and VAS at 12 Months) NDI: Neck Disability Index; VAS: Visual Analogue Scale

Outcome	Mean Rank (Zero-Profile)	Mean Rank (Plate + Graft)	U	Z	p-value
NDI	52.16	51.80	1280	-0.244	0.807
VAS	51.75	52.40	1275	-0.218	0.828

At 12 months, fusion success (Grade 1) was the most frequent outcome in both groups, with no significant difference (Table 4). Re-operations were notably absent in the zero-profile cage group but were required in a small proportion of patients with bone graft and plate, reflecting a significant difference. Adjacent segment degeneration and early postoperative complications were similar across groups, with most patients experiencing uneventful recovery and only a few developing minor issues such as dysphagia or wound infection. P-values were calculated using the chi-square test, and a p<0.05 was considered significant.

**Table 4:** Clinical Outcomes: Chi-Square Test (n, %, x2) With Categories

Outcome	Category	Group A (n=57)	Group B (n=46)	x^2^	p-value
Fusion at 12 Months	Grade 1 (Fused with remodeling & trabeculae)	37 (64.9%)	32 (69.6%)	3.653	0.301
	Grade 2 (Graft intact, not fully remodeled)	14 (24.6%)	7 (15.2%)		
	Grade 3 (Potential lucency at graft)	6 (10.5%)	5 (10.9%)		
	Grade 4 (Fusion absent, collapse/resorption)	0 (0.0%)	2 (4.3%)		
Re-operation Required	Yes	0 (0.0%)	6 (13.0%)	7.895	0.005
	No	57 (100%)	40 (87.0%)		
Adjacent Segment Degeneration	Yes	8 (14.0%)	6 (13.0%)	0.021	0.884
	No	49 (86.0%)	40 (87.0%)		
Early Post-op Complications	None	47 (82.5%)	39 (84.8%)	0.865	0.930
	Dysphagia	4 (7.0%)	3 (6.5%)		
	Wound Infection	4 (7.0%)	3 (6.5%)		
	Neurological Worsening	1 (1.8%)	1 (2.2%)		
	Hoarseness	1 (1.8%)	0 (0.0%)		

## Discussion

This study assessed the clinical and radiological outcomes of patients who underwent ACDF utilizing either a zero-profile cage or bone graft with plate fixation. Both groups exhibited notable enhancements in NDI and VAS scores at the 12-month postoperative mark. The Wilcoxon signed-rank test indicated that a majority of patients demonstrated improvement, with 78.9% in the zero-profile group and 67.4% in the plate and graft group achieving significant clinical enhancement. The findings align with prior research indicating effective pain relief and functional enhancement post-ACDF, as reported by multiple studies [5,18-20].

Between-group comparisons utilizing the Mann-Whitney U test indicated no statistically significant differences in NDI and VAS scores at 12 months (p = 0.807 and p = 0.828, respectively), suggesting that both surgical techniques yield comparable functional outcomes. This is consistent with previous studies indicating that zero-profile devices provide efficacy comparable to that of traditional plate and graft constructs, as demonstrated by Niu et al. [19] and Shin et al. [21]. Conversely, certain studies indicate that zero-profile may offer enhanced stability regarding NDI, as noted by Mustafa et al. [20], although this was not evident in our cohort, possibly due to variations in patient selection, surgical technique, and follow-up duration compared with previous studies.

Fusion rates at 12 months were comparable between groups, with Grade 1 fusion achieved in 64.9% of the zero-profile group and 69.6% of the plate and graft group (p = 0.301). This is in line with findings reported by other studies [19,20,22]. Re-operation rates were significantly higher in the plate and graft group (13.0% vs. 0%, p = 0.005), primarily due to graft-related complications. These findings support previous evidence that zero-profile constructs may reduce the risk of hardware-related complications and re-operations, as demonstrated by Nawaz et al. [23]. Early postoperative complications, including dysphagia, wound infection, neurological worsening, and hoarseness, were infrequent and comparable between groups (p = 0.930). For example, dysphagia with plate fixation was 3/46 (6.5%) and with zero-profile cages was 4/57 (7.0%). This observation contrasts with some literature reporting lower complication rates with zero-profile cages, including studies by Mustafa et al. [20], Huh et al. [22], Shin et al. [21], and Liang et al. [24]. This variation from prior studies may result from differences in sample size, patient demographics, surgical technique, and the criteria used to define complications, all of which can influence postoperative outcomes. Adjacent segment degeneration occurred in a small proportion of patients in both groups, 8 (14.0%) vs. 6 (13.0%), p = 0.884, indicating that implant choice does not significantly influence this long-term complication.

This study has several methodological strengths. It was conducted prospectively with standardized surgical techniques, uniform perioperative protocols, and validated outcome measures (NDI and VAS). All patients completed the 12-month follow-up, eliminating attrition bias. Radiological fusion was independently assessed by two experienced spine surgeons, with substantial inter-rater reliability (κ = 0.78), thereby enhancing the reliability of radiographic outcomes. Furthermore, inclusion of both single-level and two-level ACDF procedures improves the clinical applicability of the findings. The sample size exceeded the calculated requirement, thereby strengthening the statistical power to detect clinically meaningful differences.

Limitations

This study has several important limitations. The sample size was relatively small, particularly in subgroup analyses for single-level (zero-profile: n=42; plate: n=33) and two-level procedures (zero-profile: n=15; plate: n=13), limiting statistical power to detect differences. Selection bias occurred due to non-randomized allocation based on surgeon preference, potentially confounded by unmeasured patient factors such as bone quality and anatomy. Information bias resulted from a lack of blinding, which may have affected subjective outcome reporting. Confounding by unmeasured variables, including bone density, anatomical variations, and surgeon experience, was not controlled. The 12-month follow-up was insufficient for long-term outcomes, and patient-reported measures were limited to NDI and VAS scores only. External validity may be limited by single-center design, specific surgical expertise, and patient population characteristics.

## Conclusions

While functional outcomes and fusion rates were comparable between groups, the zero-profile cage demonstrated a significantly lower re-operation rate. This finding suggests a potential advantage in reducing the need for revision surgery, rather than a general reduction in postoperative complications.
